# Survivorship of the retained femoral component after revision total hip arthroplasty: A systematic review and meta-analysis

**DOI:** 10.3389/fsurg.2022.988915

**Published:** 2022-10-13

**Authors:** Hua Li, Tengfeng Zhuang, Wenrui Wu, Wenyi Gan, Chongjie Wu, Sijun Peng, Songwei Huan, Ning Liu

**Affiliations:** Department of Orthopaedics, The First Affiliated Hospital of Jinan University, Guangzhou, China

**Keywords:** retained femoral component, revision total hip arthroplasty, systematic review and meta-analysis, meta-regression, re-revision rate

## Abstract

**Objective:**

This systematic review and meta-analysis aimed to estimate re-revision rates of retained femoral components after revision of total hip arthroplasty (THA).

**Methods:**

Papers were searched in the PubMed, Embase, Web of Science, and Cochrane Library databases with predetermined keywords from January 2000 to January 2022. The studies reporting the re-revision rates of retained stems after revision THA were identified. Pooled rates of re-revision for any reason and aseptic loosening were calculated using a random-effects model or a fixed-effects model based on the results of heterogeneity assessment after the Freeman–Tukey double-arcsine transformation. A meta-regression was performed to explore potential sources of heterogeneity.

**Results:**

There were 20 studies with 1,484 hips that received the isolated cup revision with the femoral component retained. The pooled re-revision rate of retained stems was 1.75% [95% confidence interval (CI) 0.43%–3.65%]. The re-revision rate of retained stems due to aseptic loosening was 0.62% (95% CI, 0.06%–1.55%). The meta-regression showed that the fixation type (cemented or cementless) was related to the re-revision rate for any reason and the re-revision rate for aseptic loosening.

**Conclusion:**

Based on the existing evidence, the isolated cup revision with a stable stem *in situ* yields low re-revision rates. The cement status of retained stems may influence the survivorship of stems.

## Introduction

Total hip arthroplasty (THA) is one of the most effective treatments for end-stage hip disorders (1). However, despite the good clinical outcomes of THA, the revision procedure of failed THA is a heavy burden. Over 50,000 revision THA procedures are performed annually in the United States, and the frequency is estimated to double by 2030 (2). Periprosthetic osteolysis and aseptic loosening are the most common causes of failure in primary THA (3). However, the mechanism is different between the femoral stem and the acetabular cup, resulting in a linear rate of loosening for femoral components while an exponentially increasing rate for acetabular components over time (4, 5). The isolated cup loosening is reported to account for over 20% of all revision THA in the United States (6). Surgeons may encounter a loose cup with a stable stem during revision THA. In the scenario of a failed cup combined with a well-positioned and stable stem, isolated cup revision with the stem retained can avoid the risk of femoral fracture and destruction of bone stock, reduce blood loss and cost, and save operative time. A previous study of the United States National Inpatient Sample database from 2009 to 2013 investigated the epidemiology of 308,723 revision THA procedures and found that the number of isolated cup revision procedures was 44,687 (14.5%) (7). In 2019, the United Kingdom National Joint Registry demonstrated that isolated cup revision accounted for 26% (1,840/7,060) of all single-stage hip revision procedures (8)^1^. On the other hand, retaining a stem in revision may preclude the exposure of the acetabular component and compromise the restoration of hip geometry. It also carries the risk of re-revision due to the failure of the stem.

Several observational studies have assessed the outcomes of isolated cup revision with a retained stem (9–11). Most researchers recommended that the selection criteria for patients who were about to undergo an isolated cup revision should be rigorous. The stability of stems must be ensured. Chen et al. reported a minimum 5-year follow-up study of 57 retained stems after isolated cup revision and found that all acetabular and femoral components were stable (12). Kim et al. also reported an optimal survival rate of 98.9% in 227 retained stems at 30.3 years (13). However, in the study by McGonagle et al. (10), 28 of 227 retained stems (12.3%) had failed 5.1 years after the isolated cup revision. The clinical outcomes of isolated cup revision were uncertain and these studies involved a relatively small sample size. The overall survivorship of retained stems has not been well established. To our knowledge, there has been no systematic review or meta-analysis on this topic. Hence, we conducted a systematic review and meta-analysis to evaluate the fate of the retained femoral stem in revision THA.

## Materials and methods

We conducted this systematic review and meta-analysis following the Preferred Reporting Items for Systematic reviews and Meta-Analyses (PRISMA) Statement protocol (14, 15).

### Search strategy and eligibility

PubMed, Embase, Web of Science, and Cochrane Library databases were searched from January 2000 to January 2022. The search keywords were: revision AND hip AND (arthroplasty OR replacement) AND (unrevised OR retain OR isolated OR retention). We developed specific search strategies for each database and references to the identified studies were checked for potential eligibility.

We included publications that reported the outcomes of revision THA with femoral stems retained.

The following exclusion criteria were used: (1) publication year before 2000; (2) unclear etiology of index revision; (3) primary THA with oncological pathology; (4) number of retained stems of <10; (5) follow-up duration of <2 years; (6) index revision related to a debridement, antibiotics and implant retention procedure; (7) femoral stem being removed and reimplanted during operation.

Non-English language reports, case reports, conference abstracts/posters, or reviews were excluded. After dropping the duplicates, two orthopedic surgeons independently reviewed the titles and abstracts to screen potentially eligible studies. The full texts were then read independently by the same two surgeons to identify the final list of publications. If there was a disagreement, a third senior orthopedic surgeon was consulted for a final assessment and consensus.

### Data extraction

After the final list of included studies was set, data were extracted, including information on the publication, patient characteristics, follow-up duration, time intervals between primary THA and index revision, fixation type of retained stems (cemented or cementless), number of re-revision stems, number of re-revision stems due to aseptic loosening and hip function score. The primary outcome of interest was the re-revision of retained stems, and the secondary outcome was the re-revision of the retained stems due to aseptic loosening. If the necessary information could not be extracted from the original paper, we contacted the corresponding author to request additional information.

### Assessment of quality and bias

The quality of the included studies was assessed independently by the two surgeons. In this regard, the Newcastle-Ottawa Scale was used (16). Publication bias is estimated by funnel plots and Egger's test (17).

### Statistical analysis

The R package (rmeta, RRID: SCR_002270, version #4.1.3) was used for statistical analysis, with *P* < 0.05 as the threshold of statistical significance. The rates of primary and secondary outcomes with 95% confidence intervals (CIs) were pooled using the Freeman–Tukey double-arcsine transformation (18, 19). The heterogeneity was assessed using the *I*^2^ statistic and the Q test. If *I*^2^ < 50% and the *P*-value for the Q test >0.05, the studies were interpreted as minimally heterogeneous and a fixed-effects model was used for the meta-analysis. If *I*^2^ > 50% or the *P*-value for the Q test <0.05, the data were considered highly heterogeneous and a random-effects model was used. The sensitivity analysis was conducted by the leave-one-out analysis. The meta-regression was utilized to identify the potential sources of heterogeneity based on predetermined factors, including the year of publication, sample size, mean age of recruited patients, gender distribution, follow-up duration, time intervals between primary THA and index revision, and fixation type. In the first univariate model, each of the predetermined factors was analyzed individually, and the factors with a crude *P*-value less than 0.1 were extracted into the final multivariable model. If a potential factor was confirmed in the final model, subgroup analysis would be performed subsequently. Other results were presented as a descriptive summary.

## Results

### Overview of search results

**Figure 1:**
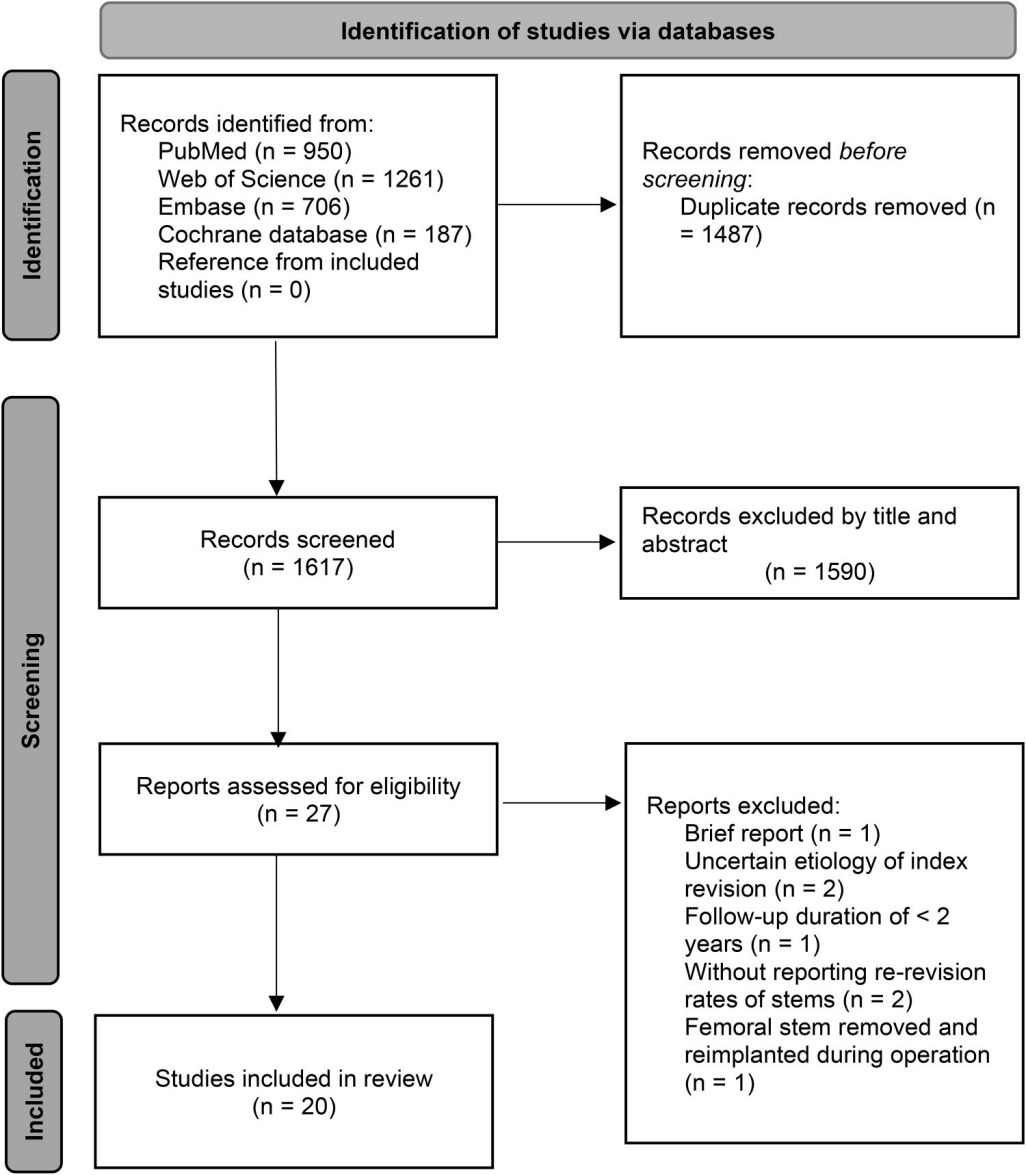
Flowchart of PRISMA.

There were 3,104 studies identified in the initial search. After deleting duplicates, the titles and abstracts of 1,617 papers were reviewed. Twenty-seven articles were assessed for eligibility by full-text reading. Finally, 20 retrospective cohort studies were included in the analysis (9, 10, 12, 13, 20–35) (Figure 1). A total of 1,484 hips underwent isolated cup revision with the stem retained. The mean follow-up duration of the studies was 12.2 years (weighted average according to the sample size) (Table 1).

**Table 1:** Characteristics of the included studies.

Authors and publication year	Number of hips	Gender (female) (%)	Mean age (y)	Fixation type	Time intervals between primary THA and index revision (y)	Follow-up duration after index revision (y)	Number of re-revision stems from total	Number of re-revision stems due to aseptic loosening	Revision for reasons other than aseptic loosening	Hip function score
Kim et al 2021	227	27.7	45.9	All CL	5	30.3	5	5	0	Postop HHS 91
Ekinci et al 2020	15	61.5	62.08	All CL	9.2	12.39	0	0	0	Preop HHS 48; Postop HHS 86
Marongiu et al 2019	30	60.0	70.6	17 CL/13 CE	9.4	11.3	0	0	0	Preop HHS 45.1; Postop HHS 85.4
Innmann et al 2019	119	NA	52	All CL	13	13	11	3	6 PFF 2 infection	NA
McGonagle et al 2015	227	66.5	68.6	All CE	15.9	6.3	28	9	8 infection 6 dislocation 3 PFF 2 others	NA
Kim et al 2015	187	53.0	47.4	161 CL/26 CE	13.7	15.6	4	4	0	Preop HHS 33; Postop HHS 88
Stathopoulos et al 2014	27	92.0	56	2 CL/25 CE	15	11	6	5	1 PFF	NA
Piolanti et al 2014	33	69.7	67	All CL	NA	36	0	0	0	Preop HHS 59; Postop HHS 88
Kim et al 2014	53	69.4	49.9	All CL	10.7	5.4	0	0	0	Preop HHS 47.5; Postop HHS 84.7
Jack et al 2013	165	52.8	65.5	All CL	12	4.8	1	0	1 thigh pain	Preop HHS 71.3; Postop HHS 91.0
Civinini et al 2012	33	54.5	69	NA	9.4	3.3	1	0	1 infection	Preop HHS 48; Postop HHS 86
Park et al 2011	69	56.7	54.9	NA	9.4	4.6	1	0	1 infection	Preop HHS 48.7; Postop HHS 86.9
Cho et al 2011	29	38.5	54.3	27 CL/2 CE	9.2	5	0	0	0	Preop HHS 56.4; Postop HHS 89.8
Fukui et al 2011	36	94.1	61	All CL	NA	6.1	0	0	0	Preop HHS 49; Postop HHS 80
He et al 2010	36	61.1	59.3	31 CL/5 CE	10.8	4.7	0	0	0	Preop HHS 57.8; Postop HHS 89.1
Lawless et al 2010	42	43.6	69	NA	NA	6.4	2	0	1 infection 1 PFF	Preop HHS 49.8; Postop HHS 80.0
Min et al 2009	24	33.3	47.1	All CL	8.2	8.3	0	0	0	Preop HHS 62; Postop HHS 92.8
Kim et al 2009	43	39.5	53	All CL	NA	6.3	0	0	0	Preop HHS 57; Postop HHS 87
Chen et al 2005	57	45.5	64	53 CL/4 CE	10	5.8	0	0	0	Preop HHS 55; Postop HHS 88
Moskal et al 2002	32	64.5	66	11 CL/22 CE	4.8	8.1	1	1	0	Preop HHS 44; Postop HHS 81

y, year; THA, total hip arthroplasty; CL, cementless; CE, cemented; HHS, Harris hip score; PFF, periprosthetic femoral fracture; NA, not available.

### Assessment of quality and bias

**Figure 2:**
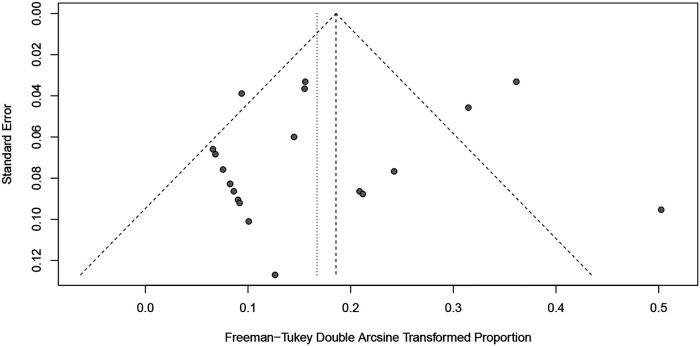
Funnel plot for the re-revision rate of retained stems for any reason.

**Figure 3:**
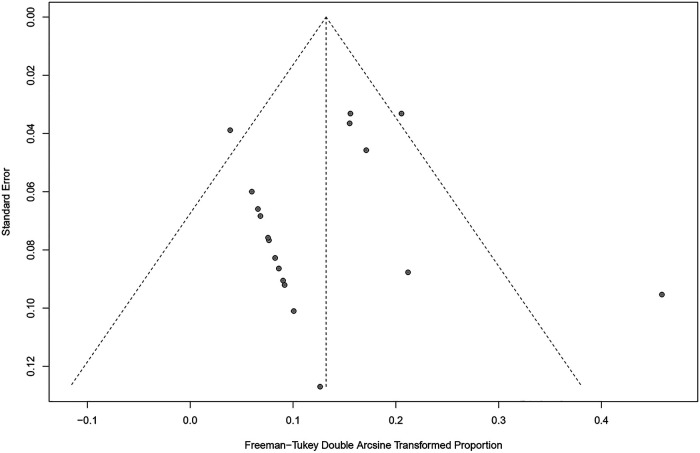
Funnel plot for the re-revision rate of retained stems for aseptic loosening.

The Newcastle-Ottawa rank for all included cohort studies was represented in Table 2. Overall, the funnel plots for the primary outcome and the secondary outcome showed no evidence of possible publication bias (Figures 2, 3), which was also consistent with the formal test (Egger's test, *P* for the primary outcome = 0.2511; *P* for the secondary outcome = 0.4440).

**Table 2:** Quality assessment of the included studies based on the Newcastle-Ottawa scale.

Authors and publication year	Selection				Comparability	Outcome			Total
	Representativeness of the exposed cohort	Selection of the nonexposed cohort	Ascertainment of exposure	Absence of the outcome of interest at the start		Assessment of outcome	Adequate length of follow-ups	Accuracy of follow-up cohorts	
Kim et al 2021	*	—	*	*	—	*	*	*	6
Ekinci et al 2020	*	—	*	*	—	*	*	—	5
Marongiu et al 2019	*	—	*	*	—	*	*	*	6
Innmann et al 2019	*	—	*	*	—	*	*	*	6
McGonagle et al 2015	*	—	*	*	—	*	*	*	6
Kim et al 2015	*	—	*	*	—	*	*	*	6
Stathopoulos et al 2014	*	—	*	*	—	*	*	*	6
Piolanti et al 2014	*	—	*	*	—	*	*	*	6
Kim et al 2014	*	—	*	*	—	*	*	*	6
Jack et al 2013	*	—	*	*	—	*	*	*	6
Civinini et al 2012	*	—	*	*	—	*	*	*	6
Park et al 2011	*	—	*	*	—	*	*	*	6
Cho et al 2011	*	—	*	*	—	*	*	*	6
Fukui et al 2011	*	—	*	*	—	*	*	*	6
He et al 2010	*	—	*	*	—	*	*	*	6
Lawless et al 2010	*	—	*	*	—	*	*	*	6
Min et al 2009	*	—	*	*	—	*	*	*	6
Kim et al 2009	*	—	*	*	—	*	*	*	6
Chen et al 2005	*	—	*	*	—	*	*	*	6
Moskal et al 2002	*	—	*	*	—	*	*	*	6

### Primary outcome

**Figure 4:**
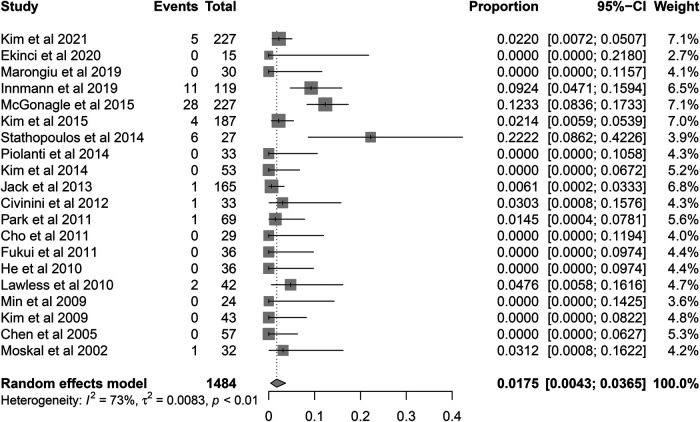
Forest plot for the re-revision rate of retained stems.

**Figure 5:**
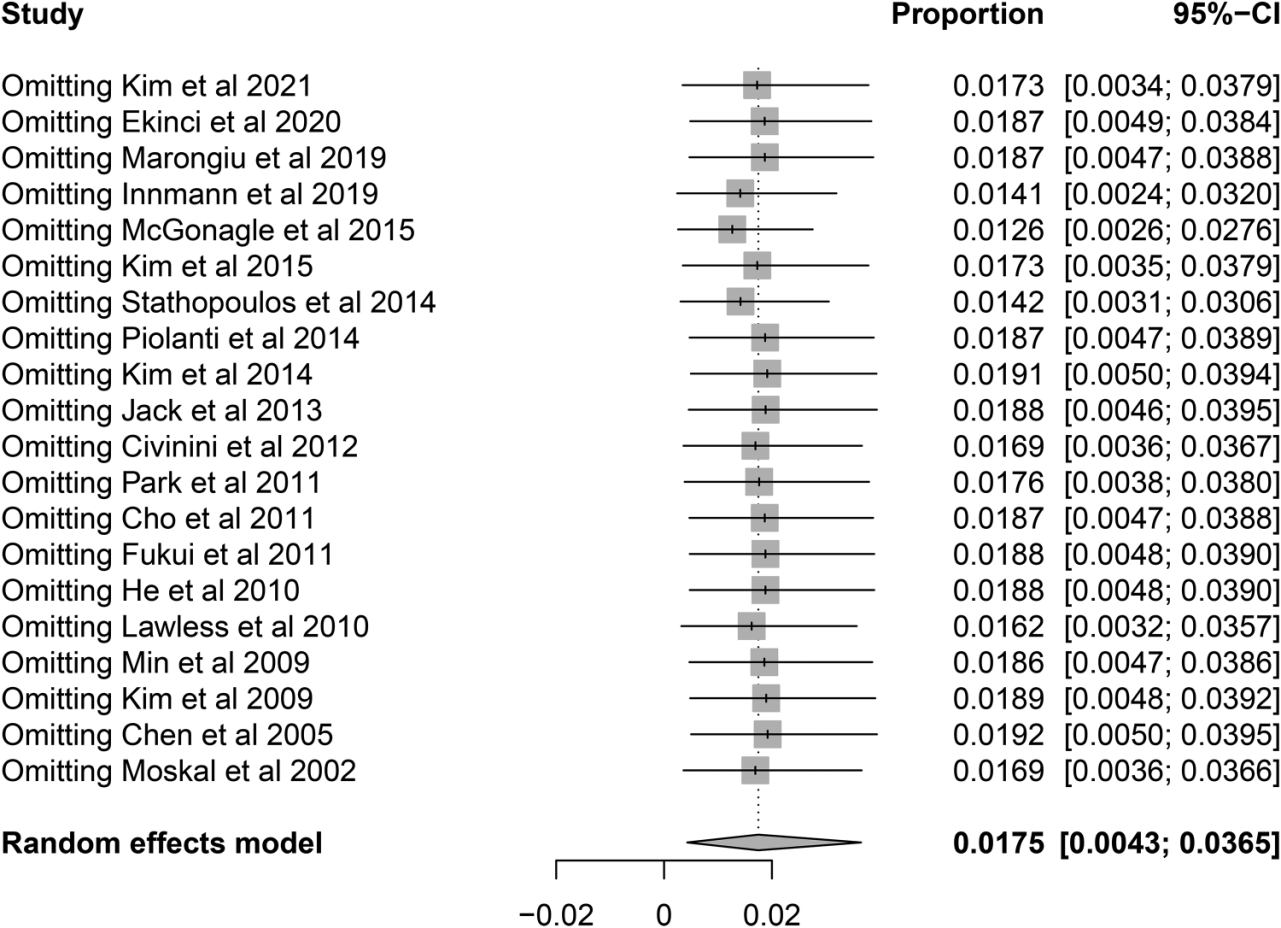
Sensitivity analysis for the re-revision rate of retained stems using leave-one-out analysis.

**Figure 6:**
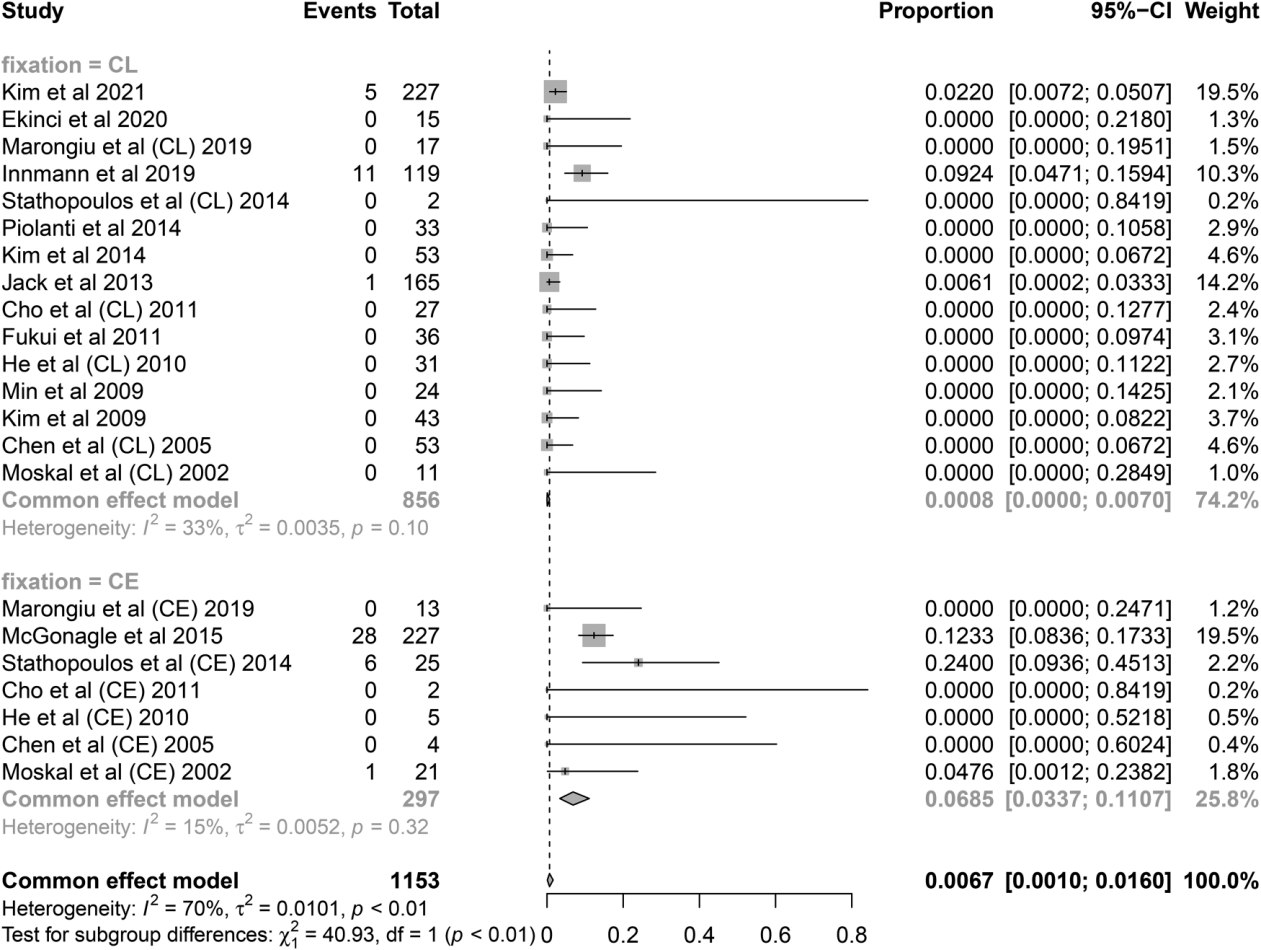
Subgroup analysis of re-revision rate by fixation type. CL, cementless; CE, cemented.

A total of 60 re-revision cases of retained stems for any reason were observed. The pooled re-revision rate was 1.75% (95% CI 0.43%–3.65%) with relatively high heterogeneity (*I*^2^ = 72.3%, *P* < 0.001) (Figure 4). The results were robust to the leave-one-out analysis (Figure 5). The meta-regression was performed to explore the potential sources of heterogeneity. At the first univariate step, fixation type and time intervals between primary THA and index revision were extracted with a crude *P*-value less than 0.1. In the next multiple regression model, the fixation type was finally identified as the only potential factor (Table 3). The subsequent subgroup analysis based on the fixation type showed that the re-revision rate was 0.08% (95% CI, 0%–0.7%; heterogeneity, *I*^2^ = 33%, *P* = 0.10) in cementless stems while that was 6.9% (95% CI, 3.4%–11.1%; heterogeneity, *I*^2^ = 15%, *P* = 0.32) in cemented stems. The inter-subgroup difference was of statistical significance (*P* < 0.001) (Figure 6).

**Table 3:** Results of the meta-regression for re-revision rate for any reason.

Parameters	Crude coefficient (95% CI)	Crude *P*	Adjusted coefficient (95% CI)	Adjusted *P*
Publication year	0.0055 (−0.0053 to 0.0163)	0.3190	—	—
Mean age	0.0014 (−0.0048 to 0.0077)	0.6524	—	—
Gender (female)	0.2139 (−0.0838 to 0.5116)	0.1590	—	—
Number of hips	0.0004 (−0.0003 to 0.0011)	0.2408	—	—
Follow-up term after the index revision	−0.0001 (−0.0062 to 0.0060)	0.9772	—	—
Time intervals between primary THA and index revision	0.0187 (0.0020–0.0355)	0.0280	0.0092 (−0.0075 to 0.0260)	0.2788
Fixation type	−0.2508 (−0.3763 to −0.1253)	<0.0001	−0.2065 (−0.3645 to −0.0484)	0.0105

CI, confidence interval; THA, total hip arthroplasty.

### Secondary outcome

**Figure 7:**
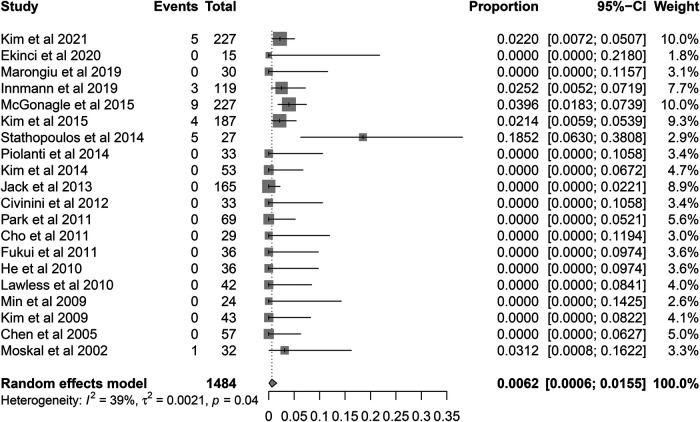
Forest plot for the re-revision rate of retained stems for aseptic loosening.

**Figure 8:**
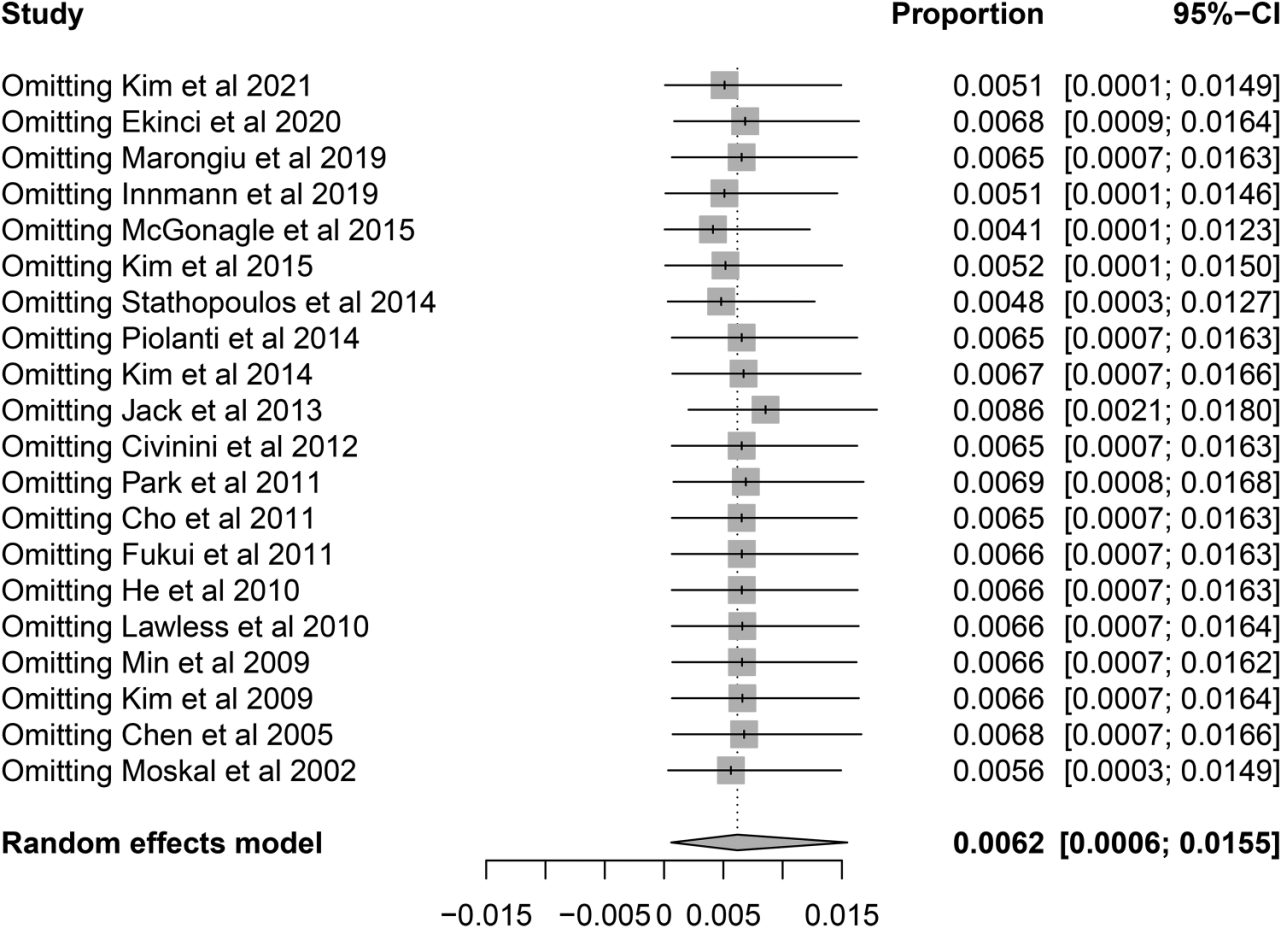
Sensitivity analysis for the re-revision rate of retained stems for aseptic loosening using leave-one-out analysis.

Among the 60 re-revision stems, a total of 27 cases (45%) were reported due to aseptic loosening. Pooled analysis showed that the rate of re-revision due to aseptic loosening was 0.62% (95% CI, 0.06%–1.55%) with a moderate heterogeneity (*I*^2^ = 38.8%, *P* = 0.0398) (Figure 7). The results were also robust to the leave-one-out analysis (Figure 8). The univariate meta-regression model also showed that the fixation type was the only potential source of heterogeneity (Table 4).

**Table 4:** Results of the meta-regression for re-revision rate for aseptic loosening.

Parameters	Crude coefficient (95% CI)	Crude *P*
Publication year	0.0045 (−0.0028 to 0.0119)	0.2264
Mean age	−0.0012 (−0.0054 to 0.0030)	0.5760
Gender	0.1585 (−0.0752 to 0.3923)	0.1838
Number of hips	0.0002 (−0.0002 to 0.0007)	0.2602
Follow-up term after the index revision	0.0020 (−0.0020 to 0.0059)	0.9722
Time intervals between primary THA and index revision	0.0075 (−0.0052 to 0.0202)	0.2458
Fixation type	−0.1425 (−0.2355 to −0.0496)	0.0027

CI, confidence interval; THA, total hip arthroplasty.

## Discussion

This is the first systematic review and meta-analysis to analyze the re-revision rates of unrevised femoral components during revision THA. We found that the retained stems after isolated cup revision represented favorable results with a pooled re-revision rate of 1.75% for any reason and 0.62% for aseptic loosening, respectively. Furthermore, our data indicated that revision THA with cemented stems retained might have a higher re-revision rate than those with cementless stems retained.

Our research is of clinical relevance for surgeons in deciding whether to remove or retain a stable stem when facing the common dilemma that only the cup is loosened as the survival rates of cups are much lower when compared with stems. Retaining a stable stem is an attractive option for the obvious advantages such as less surgical time, lower risk of intraoperative fracture, less impairment on the bone stock of the proximal femur, and less blood loss and cost. Poon and Lachiewicz (36) reported that, when compared with revision of both the cups and stems, the blood loss was 52% less and the surgical time was 35% shorter in isolated cup revision. Lim et al. compared the outcomes of isolated cup revision with a matched total revision group and found that the hip function and survivorship were similar between the two groups (37). Nevertheless, removing a well-fixed stem can facilitate the exposure and the selection of type and size of the revision prostheses and enhance the longevity of both cups and stems by the newer-generation implants, which is called “fresh start” (23, 36, 38). The present review aggregated the existing evidence on the controversy to evaluate the survivorship of the retained stable stems during revision THA.

We found that, during a relatively considerable follow-up term, the retained stems showed high survivorship with a re-revision rate of 1.75%. The two highest re-revision rates of the studies were 22.2% by Stathopoulos et al. (23) and 12.3% by McGonagle et al. (10), respectively, and they were within the published range. There were two previous studies with a sample size of over 1,000 revision THAs and a follow-up of over 10 years. Philpott et al. reported a stem survivorship of 77% in 1,176 revision THAs with a minimum 10-year follow-up (39). Springer et al. reported an overall survivorship of 82% at 10 years of 1,100 revision THAs (40). Our data on the re-revision rate of stems due to aseptic loosening (0.62%) was similar to those reported by Hasegawa et al. (2.2%) (41) and Imbuldeniya et al. (0.7%) (42). These results revealed that isolated acetabular revision was a reliable procedure in the presence of a well-fixed stem. We also noted that the pooled re-revision rates seemed to be lower than those reported by the large-sample cross-sectional studies, which might be partly due to the discreet selection of patients. Many studies emphasized that the accurate and secure fixation and proper alignment of a stem was the prerequisite for stem retention. Surgeons should assess the stem stability not only by radiographs but also intraoperatively. Ekinci et al. and Cho et al. confirmed the stability of the stem by conducting traction and rotational forces on stems and detecting the fine movements between stems and adjacent host bone during the revision procedure (20, 29). Hernigou et al. recommended that the osteolysis around the retained stems should be less than 10 cm^2^ to achieve longer survivorship (43). Kim et al. and Innmann et al. further restricted the indication that the osteolysis should be in Gruen zones 1 and/or 7 (13, 21). Cautiously screening patients meeting the indication could improve the survivorship of the surgeries. In addition, the pooled calculation of re-revision rates was based on retrospective data and thus could not reflect the total number of hips undergoing the isolated cup revision with the stem retained. Therefore, further studies with longer follow-ups, larger sample sizes, and higher levels of evidence are needed to demonstrate a more accurate survival rate of retained stems.

Our meta-regression reflected that fixation type would influence the fate of the retained stems. The retained stems with cemented fixation might be associated with a higher risk of re-revision than those with cementless fixation. When compared to the cementless technique, cementing may increase the risk of periprosthetic osteolysis due to cement particles (44). Emerson et al. investigated the radiographic findings of cemented and cementless stems in 180 primary THA cases and found a higher incidence of osteolysis in cemented stems (45). The authors stated that cementless stems were more resistant to osteolysis. Several meta-analyses have investigated the influence of cement status on stem survivorship. Toci et al. included seven studies and found that cementless stems were related to a lower revision rate (5.53% vs. 8.91%) when compared with cemented stems in primary THA, but the difference was not statistically significant (46). Another meta-analysis suggested that older patients might benefit more from cemented fixation while younger patients benefit more from cementless fixation in primary THA (47). Due to a paucity of publications, no study has so far compared the outcome of a cemented retained stem with that of a cementless retained stem directly. The implication of our results should be validated by future comparative studies. Other factors that may affect the outcomes of retained stems during revision THA have also been discussed. The re-revision rate reported by Stathopoulos et al. was the highest among the included studies. The majority of failed retained stems (90%) in their studies belonged to patients with developmental dysplasia of the hip (23). The authors attributed the relatively high failure rate of retained stems to the poor pre-existing femoral bone quality. Several studies have identified that the rate of hip instability or dislocation after isolated cup revision might be associated with surgical approaches (48, 49). Moskal et al. recommended a standard modified direct lateral approach to avoid excessive soft tissue release (35).

Several limitations should be noted in this research. First, the methodology contained the bias of possibly inevitably missing relevant studies. However, we identified and included all eligible studies on the re-revision rates of retained stems from main databases. Second, the heterogeneity of the pooled analysis was relatively high. We, therefore, performed a meta-regression to explore potential sources and found that the type of fixation could influence the results. The subsequent subgroup analysis also reduced the heterogeneity. Third, though the stratification based on fixation type showed a positive result in the comparison between cemented and cementless stems, the statistical analysis might not be sufficiently rigorous due to the relatively small sample size of failed stems. Fourth, the data were from retrospective studies rather than the registers or prospective studies. The pooled analysis could not reflect the cross-sectional panorama of unrevised stems after revision THA, which would introduce a bias in the estimation of re-revision rates. Thus, the results should be interpreted with caution.

## Conclusion

Based on the available evidence, the isolated cup revision while retaining a stable stem *in situ* yields low re-revision rates. Retained stems with cemented fixation might have a higher risk of re-revision than those with cementless fixation.

## Data Availability

The original contributions presented in the study are included in the article/Supplementary Material, further inquiries can be directed to the corresponding authors.

## Footnotes

^1^ https://reports.njrcentre.org.uk/hips-revision-procedures-components/H21v1NJR?reportid=471D9229-25EC-4D5F-B220-425F3DCD3518/defaults=DC__Reporting_Period__Date_Range=%22MAX%22,J__Filter__Calendar_Year=%22MAX%22,H__Filter__Joint=%22Hip%22.
